# Development of a lung perfusion automated quantitative model based on dual-energy CT pulmonary angiography in patients with chronic pulmonary thromboembolism

**DOI:** 10.1186/s13244-025-02067-6

**Published:** 2025-08-18

**Authors:** Linfeng Xi, Jianping Wang, Anqi Liu, Yifei Ni, Jie Du, Qiang Huang, Yishan Li, Jing Wen, Hongyi Wang, Shuai Zhang, Yunxia Zhang, Zhu Zhang, Dingyi Wang, Wanmu Xie, Qian Gao, Yong Cheng, Zhenguo Zhai, Min Liu

**Affiliations:** 1 China-Japan Friendship Hospital, Capital Medical University, Beijing, China; 2 National Center for Respiratory Medicine, State Key Laboratory of Respiratory Health and Multimorbidity, National Clinical Research Center for Respiratory Diseases, Institute of Respiratory Medicine, Chinese Academy of Medical Sciences, Department of Pulmonary and Critical Care Medicine, Center of Respiratory Medicine, China-Japan Friendship Hospital, Beijing, China; 3 Department of Radiology, China-Japan Friendship Hospital, Beijing, China; 4 Chinese Academy of Medical Sciences and Peking Union Medical College, Beijing, China; 5 The First Clinical Medical College, Shanxi Medical University, Taiyuan, China; 6 National Center for Respiratory Medicine, State Key Laboratory of Respiratory Health and Multimorbidity, National Clinical Research Center for Respiratory Diseases, Institute of Respiratory Medicine, Chinese Academy of Medical Sciences, Data and Project Management Unit, Institute of Clinical Medical Sciences, China-Japan Friendship Hospital, Beijing, China; 7 Beijing University of Chemical Technology, Beijing, China

**Keywords:** Chronic pulmonary thromboembolism, Dual-energy computed tomography pulmonary angiography, Artificial intelligence, U-Net, Adaptive thresholding algorithm

## Abstract

**Objective:**

To develop PerAIDE, an AI-driven system for automated analysis of pulmonary perfusion blood volume (PBV) using dual-energy computed tomography pulmonary angiography (DE-CTPA) in patients with chronic pulmonary thromboembolism (CPE).

**Materials and methods:**

In this prospective observational study, 32 patients with chronic thromboembolic pulmonary disease (CTEPD) and 151 patients with chronic thromboembolic pulmonary hypertension (CTEPH) were enrolled between January 2022 and July 2024. PerAIDE was developed to automatically quantify three distinct perfusion patterns—normal, reduced, and defective—on DE-CTPA images. Two radiologists independently assessed PBV scores. Follow-up imaging was conducted 3 months after balloon pulmonary angioplasty (BPA).

**Results:**

PerAIDE demonstrated high agreement with the radiologists (intraclass correlation coefficient = 0.778) and reduced analysis time significantly (31 ± 3 s vs. 15 ± 4 min, *p* < 0.001). CTEPH patients had greater perfusion defects than CTEPD (0.35 vs. 0.29, *p* < 0.001), while reduced perfusion was more prevalent in CTEPD (0.36 vs. 0.30, *p* < 0.001). Perfusion defects correlated positively with pulmonary vascular resistance (ρ = 0.534) and mean pulmonary artery pressure (ρ = 0.482), and negatively with oxygenation index (ρ = –0.441). PerAIDE effectively differentiated CTEPH from CTEPD (AUC = 0.809, 95% CI: 0.745–0.863). At the 3-month post-BPA, a significant reduction in perfusion defects was observed (0.36 vs. 0.33, *p* < 0.01).

**Conclusion:**

CTEPD and CTEPH exhibit distinct perfusion phenotypes on DE-CTPA. PerAIDE reliably quantifies perfusion abnormalities and correlates strongly with clinical and hemodynamic markers of CPE severity.

**Trial registration:**

ClinicalTrials.gov, NCT06526468. Registered 28 August 2024- Retrospectively registered, https://clinicaltrials.gov/study/NCT06526468?cond=NCT06526468&rank=1.

**Critical relevance statement:**

PerAIDE is a dual-energy computed tomography pulmonary angiography (DE-CTPA) AI-driven system that rapidly and accurately assesses perfusion blood volume in patients with chronic pulmonary thromboembolism, effectively distinguishing between CTEPD and CTEPH phenotypes and correlating with disease severity and therapeutic response.

**Key Points:**

- Right heart catheterization for definitive diagnosis of chronic pulmonary thromboembolism (CPE) is invasive.
- PerAIDE-based perfusion defects correlated with disease severity to aid CPE-treatment assessment.
- CTEPH demonstrates severe perfusion defects, while CTEPD displays predominantly reduced perfusion.
- PerAIDE employs a U-Net-based adaptive threshold method, which achieves alignment with and faster processing relative to manual evaluation.

**Graphical Abstract:**

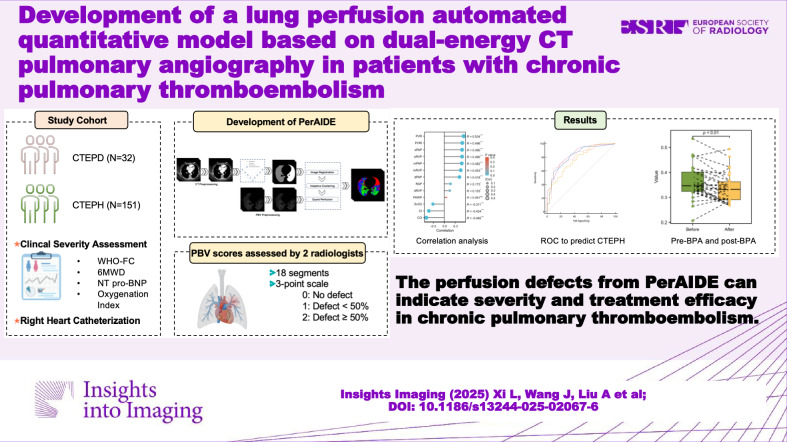

## Introduction

Chronic thromboembolic pulmonary hypertension (CTEPH) and chronic thromboembolic pulmonary disease (CTEPD) are symptomatic manifestations of chronic pulmonary thromboembolism (CPE), differentiated primarily by the presence or absence of resting pulmonary hypertension (PH) [1]. Recent evidence suggests that CTEPD develops in approximately 28% of patients following an episode of acute pulmonary embolism (APE) [2], significantly impacting both quality of life and long-term clinical outcomes [3, 4]. Although the incidence of CTEPH among APE survivors is relatively low at 2.7% [5], it remains the most severe chronic complication. Diagnosis and treatment are often delayed due to nonspecific symptoms and the invasive nature of right heart catheterization (RHC), which is required for definitive diagnosis.

Computed tomography pulmonary angiography (CTPA) plays a central role in the evaluation of CPE. The use of dual-energy computed tomography pulmonary angiography (DE-CTPA) is gaining prominence owing to its ability to simultaneously visualize pulmonary emboli and assess perfusion through iodine mapping, thereby improving the overall diagnostic accuracy in CPE [6, 7]. Importantly, radiation exposure during DE-CTPA is comparable to that of a traditional ventilation/perfusion (V/Q) scan [8]. DE-CTPA achieves high diagnostic accuracy in detecting CPE and shows good concordance with V/Q scanning [9, 10]. However, variability in the interpretation of iodine perfusion maps across radiologists introduces subjectivity, underscoring the need for standardized, objective evaluation tools.

Artificial intelligence (AI) and adaptive algorithms have shown substantial promise in various radiologic applications, such as segmenting infarct cores and ischemic penumbra in acute ischemic stroke and detecting post-treatment hemorrhagic transformation [11, 12]. Inspired by these advances, we hypothesize that similar AI-based techniques can facilitate quantitative, noninvasive assessment of pulmonary perfusion blood volume (PBV) on DE-CTPA in patients with CPE. Moreover, the association between quantitative perfusion metrics and clinical parameters—including hemodynamics and disease severity—in both CTEPD and CTEPH remains underexplored. Therefore, the objectives of this study are as follows: (1) to develop an AI-powered pulmonary perfusion quantification system based on DE-CTPA, termed PerAIDE; (2) to compare perfusion characteristics between patients with CTEPD and CTEPH; and (3) to investigate the correlations between PerAIDE-derived perfusion parameters and clinical data, and evaluate the potential of these metrics to reflect disease severity and treatment response in CTEPD and CTEPH.

## Materials and methods

### Study design and cohort

**Fig. 1:**
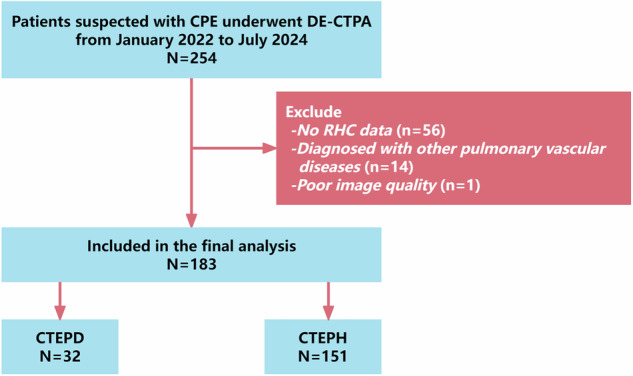
Flowchart of the study. CPE, chronic pulmonary thromboembolism; DE-CTPA, dual-energy computed tomography pulmonary angiography; RHC, right heart catheterization; CTEPD, chronic thromboembolic pulmonary disease; CTEPH, chronic thromboembolic pulmonary hypertension

This was a prospective, single-center observational cohort study conducted between January 2022 and July 2024. The study received approval from the institutional review board (approval number: 2022-KY-240) and was performed in accordance with the Declaration of Helsinki. Patients with suspected CPE who presented to our institution during the study period were consecutively enrolled after providing written informed consent. Patients were eligible for inclusion if they were older than 18 years and had undergone both DE-CTPA and RHC within a 48-h window. The exclusion criteria included poor imaging quality due to suboptimal pulmonary artery contrast enhancement or severe respiratory motion artifacts, the presence of other pulmonary vascular diseases including APE, Takayasu arteritis, fibrosing mediastinitis, pulmonary artery sarcoma, or other forms of PH, and a history of pulmonary thromboendarterectomy prior to CTPA (Fig. 1). The diagnosis of CPE was based on established criteria [13], requiring confirmation of chronic thromboembolic obstruction on either CTPA or a V/Q scan after a minimum of 3 months of standardized anticoagulation therapy.

### Clinical assessments

Comprehensive clinical assessments were performed for each participant. Demographic data, including age, sex, and body mass index (BMI), were recorded. Clinical characteristics, such as comorbidities, laboratory findings, World Health Organization functional class (WHO-FC), and 6-min walk distance (6MWD) were documented. A multidisciplinary team of experts established the final diagnosis of either CTEPD or CTEPH through consensus, based on a combination of clinical presentation, imaging evidence of organized fibrotic obstructions and perfusion abnormalities, and hemodynamic data obtained via RHC [13].

### RHC

All patients underwent RHC, which was performed via either the right internal jugular or femoral vein using a 6F Swan-Ganz catheter (Bioptimal). The following hemodynamic parameters were measured: mean pulmonary artery pressure (mPAP), mean right ventricular pressure, mean right atrial pressure, systolic pulmonary artery pressure (sPAP), systolic right ventricular pressure (sRVP), pulmonary arterial wedge pressure, pulmonary vascular resistance (PVR), pulmonary vascular resistance index and mixed venous oxygen saturation (SvO_2_). Cardiac output (CO) and cardiac index (CI) were calculated using the Fick method. Patients were classified into the CTEPH group if mPAP exceeded 20 mmHg, and into the CTEPD group otherwise. Therapeutic efficacy was evaluated at a 3-month follow-up.

### DE-CTPA scan protocol

All DE-CTPA examinations were performed using a dual-source computed tomography (CT) scanner (Siemens Healthineers) equipped with dual-energy acquisition at 100 kVp and Sn140 kVp. Scanning parameters included the use of CARE Dose4D, a rotation time of 0.28 s, collimation of 64 × 0.6 mm, and a pitch of 0.55. Intravenous contrast was administered using a total of 48 mL of iopromide at a flow rate of 4.0 mL/s, followed by a 20 mL saline flush. Automated bolus tracking was employed by placing a region of interest in the main pulmonary artery, with image acquisition initiated after a 5-s delay once a 100 HU threshold was reached. Whole-chest scanning in the craniocaudal direction was completed during a single breath-hold. Images were reconstructed with a 1.5-mm slice thickness at 1.0-mm intervals. PBV maps were generated using the SyngoVia post-processing platform (Siemens Healthineers), specifically designed for DE-CTPA image analysis. The CTDvol was 4.31 mGy/cm.

### Development of PerAIDE, a pulmonary perfusion AI automatic quantification analysis system based on DE-CTPA

#### Data preprocessing

Anonymized DE-CTPA Digital Imaging and Communications in Medicine (DICOM) data underwent standardized preprocessing. First, noise reduction was performed using adaptive bilateral filtering to preserve edge details in both CT and PBV images [14]. Second, intensity normalization was applied to standardize grayscale ranges, which is critical for cross-patient comparisons of PBV perfusion. Third, geometric correction was performed to address respiratory and motion artifacts through connected component analysis, in which components smaller than 5% of the largest connected region were removed. Fourth, contrast enhancement was conducted using lung-specific contrast-limited adaptive histogram equalization (CLAHE) [15] with a clip limit of 2.0 and an 8 × 8 tile grid, enhancing structural clarity in CT images and improving perfusion contrast in PBV maps.

#### Image segmentation

**Fig. 2:**
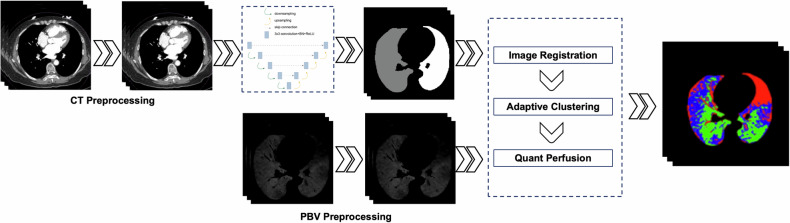
The pipeline of pulmonary perfusion artificial intelligence automatic quantification analysis system (PerAIDE) based on dual-energy CT pulmonary angiography. First, PerAIDE was used to preprocess the CT images and PBV images. Then, a U-Net architecture was used to undertake lung segmentation. Next, rigid registration was performed between the CT images and PBV images. After image registration, we applied an adaptive thresholding algorithm to the registered PBV image pixels within the segmented lung regions. For visualization, we generate color-coded overlay maps on the original CT images by using a carefully selected color scheme to represent different perfusion categories: red for perfusion defects, blue for reduced perfusion, and green for normal areas. The quantification outputs provide volumetric measurements (absolute values and percentages) of the three distinct color-coded regions relative to the total lung volume. PerAIDE, pulmonary perfusion artificial intelligence automatic quantification analysis system based on DE-CTPA; PBV, perfusion blood volume

Lung segmentation was done using an optimized U-Net architecture (Fig. 2), consisting of an encoder-decoder structure with skip connections. The encoder included four downsampling blocks, each comprising two 3 × 3 convolutional layers followed by batch normalization, ReLU activation, and 2 × 2 max pooling with a stride of 2. The number of feature channels increased sequentially from 64 to 512. A dropout layer with a rate of 0.5 was applied at the bottleneck for regularization. The decoder mirrored this structure, employing 2 × 2 transposed convolutions to halve the number of channels and double the spatial resolution, with skip connections concatenating corresponding encoder features. The final layer was a 1 × 1 convolution with sigmoid activation, generating a probability map for segmentation. The network was trained on 512 × 512 CT slices with a batch size of 16 using the Adam optimizer (learning rate = 1e^−4^) and a hybrid loss function combining Dice loss and binary cross-entropy. This segmentation preserved anatomical accuracy, enabling precise co-registration of PBV maps and serving as a crucial input for downstream lung function assessment.

#### Quantification and visualization

**Fig. 3:**
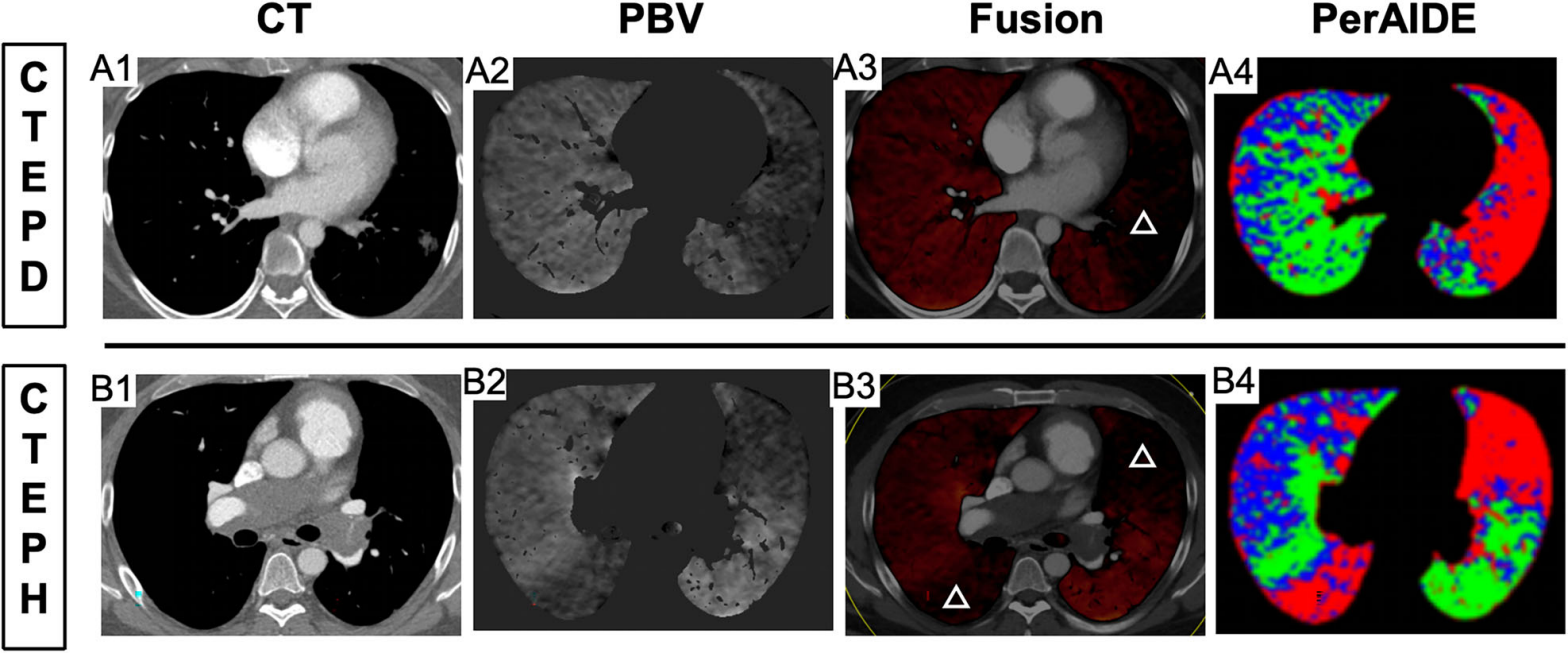
Representative images of CTEPD and CTEPH. **A1–A4** refer to images from the same slice of a typical CTEPD patient. **B1–B4** refer to images from the same slice of a typical CTEPH patient. (**A1**) The CT image of CTEPD; (**A2**) The lung PBV image of CTEPD; (**A3**) The CT/PBV fusion image of CTEPD; (**A4**) The final image generated by the PerAIDE system of CTEPD. (**B1**) The CT image of CTEPH; (**B2**) The lung PBV image of CTEPH; (**B3**) The CT/PBV fusion image of CTEPH; (**B4**) The final image generated by the PerAIDE system of CTEPH. White triangles in **A3** and **B3** represent the perfusion defects area. In **A4** and **B4**, the perfusion defects are areas in the pulmonary vasculature where there is a total absence of blood flow (red regions). Areas with decreased perfusion have reduced blood flow intensity (blue regions), but not a complete absence. Normally perfused areas have entirely normal blood flow (green regions). CTEPD, chronic thromboembolic pulmonary disease; CTEPH, chronic thromboembolic pulmonary hypertension; PBV, perfusion blood volume; PerAIDE, pulmonary perfusion artificial intelligence automatic quantification analysis system based on DE-CTPA

Quantitative analysis was performed on the registered CT and PBV images (Fig. 2). Rigid registration was employed to ensure accurate anatomical alignment between CT and PBV datasets. Lung regions were categorized into three perfusion states—perfusion defects, reduced perfusion, and normal perfusion—using adaptive thresholding based on local intensity distributions, ensuring robustness against heterogeneous patterns. Neighborhood spatial smoothing was applied to refine boundaries and minimize false-positive misclassifications. Quantitative metrics were then computed, including the absolute volume and relative percentage of each perfusion category in relation to the total lung volume. For visual representation, we generate color-coded overlay maps on the original CT images, with distinct color codes used to differentiate perfusion categories (Fig. 2). Figure 3 illustrates representative CT images, PBV maps, CT-PBV fusion images and  automated quantitative analysis results generated by the PerAIDE system from typical CTEPD and CTEPH cases. Perfusion defects, shown in red (A4 and B4), represent regions with a complete absence of pulmonary blood flow. Areas with reduced perfusion, shown in blue, reflect diminished but not absent perfusion. Green-coded regions represent normally perfused areas. Further details of the workflow are provided in Supplementary Materials (Part 1).

#### Manual evaluation

Manual evaluation was independently conducted by two board-certified chest radiologists, blinded to clinical data. They assessed 18 lung segments on axial PBV images using a standardized 3-point scoring system [16]: 0 indicating no perfusion defect, 1 for defects involving less than 50% of the segmental volume, 2 for defects involving 50% or more (Fig. S1). All evaluations were performed using axial color-coded PBV maps, in which black and red regions indicated hypoperfusion and normal perfusion, respectively. Pseudo-defects resulting from cardiac motion or contrast artifacts in the great vessels were excluded from the analysis. Any discrepancies between radiologists were resolved by consensus, and final scores were derived by summing all segmental scores. In addition to PBV scoring, radiologists’ interpretation time was recorded to enable a quantitative comparison of efficiency between manual evaluation and the PerAIDE system.

### Statistical analysis

All statistical analyses were performed using R version 4.4.2 and MedCalc version 20.0.22. Continuous variables were expressed as mean ± standard deviation or median with interquartile range, while categorical variables were summarized as frequencies and percentages. Paired *t*-tests were used to compare pre- and post-treatment parameters for normally distributed data. It is important to note that manual PBV scoring evaluates only the extent of perfusion defects and does not account for intensity variations. To assess the accuracy of PerAIDE-derived parameters, regions of reduced perfusion and perfusion defects identified by the system were combined and defined as malperfused regions. The percentage of malperfused lung volume was then compared with manual PBV scores for agreement analysis. Interobserver agreement was quantified using the intraclass correlation coefficient (ICC) based on a two-way mixed-effects model, with thresholds defined as follows: < 0.40 (poor), 0.40–0.59 (fair), 0.60–0.74 (good), ≥ 0.75 (excellent). Spearman’s correlation coefficients, with strengths categorized as strong (|ρ| ≥ 0.7), moderate (0.5–0.7), weak (0.3–0.5), and negligible (< 0.3). Diagnostic performance was assessed via receiver operating characteristic (ROC) curve analysis, with the area under the curve (AUC) serving as the primary metric. A *p*-value of less than 0.05 was considered statistically significant.

## Results

### Patient characteristics

Among 254 patients who underwent DE-CTPA between January 2022 and July 2024, 183 patients (32 with CTEPD and 151 with CTEPH) met the inclusion criteria. The exclusions included 56 patients without RHC data, 14 diagnosed with other pulmonary vascular diseases, and 1 with suboptimal image quality. The study flowchart is presented in Fig. 1. Baseline characteristics and clinical data are detailed in Table 1. The median patient age was 58 years, with males comprising 49.18% (*n* = 90) of the cohort. A history of venous thromboembolism (VTE) was present in the majority of patients (*n* = 152, 83.06%), while only 8 patients had a history of cancer. More than half (*n* = 116, 63.39%) had comorbid cardiopulmonary diseases. Most patients were classified as WHO-FC II, with a median 6MWD of 430 m.

**Table 1:** The demographic and clinical characteristics of patients in this study

Characteristics	All patients (*n* = 183)	CTEPD (*n* = 32)	CTEPH (*n* = 151)	*p*-value
Age, median years (range)	58.00 (46.50, 67.00)	48.50 (32.50, 62.00)	60.00 (48.00, 67.00)	0.004*
Sex, male (*n*, %)	90 (49.18)	18 (56.25)	72 (47.68)	0.379
BMI, kg/m^2^	24.03 (21.88, 26.11)	24.88 (21.94, 27.34)	23.93 (21.88, 25.84)	0.176
Smoker, *n* (%)	47 (25.68)	6 (18.75)	41 (27.15)	0.323
Cardiopulmonary comorbidity, *n* (%)	116 (63.39)	15 (46.88)	101 (66.89)	0.033*
Disease duration, days	337.00 (128.50, 723.00)	216.50 (114.00, 429.50)	376.00 (143.50, 776.50)	0.027*
History of VTE, *n* (%)	152 (83.06)	29 (90.62)	123 (81.46)	0.209
PTE, *n* (%)	156 (85.25)	29 (90.62)	127 (84.11)	0.503
DVT, *n* (%)	72 (39.34)	18 (56.25)	54 (35.76)	0.031*
Cancer, *n* (%)	8 (4.37)	2 (6.25)	6 (3.97)	0.923
WHO-FC I/II/III/IV, *n* (%)	31/108/42/2	19/12/1/0	12/96/41/2	< 0.001*
6MWD, m	430.00 (368.00, 485.00)	515.00 (445.00, 550.00)	421.00 (365.25, 472.25)	< 0.001*
PaO_2_, mmHg	68.50 (58.25, 81.00)	87.00 (80.00, 93.40)	66.00 (57.00, 74.80)	< 0.001*
Oxygenation index	315.76 (271.43, 378.14)	414.29 (371.43, 444.76)	304.76 (261.90, 342.86)	< 0.001*
D-dimer, mg/L	0.19 (0.13, 0.33)	0.14 (0.10, 0.22)	0.20 (0.14, 0.37)	0.015*
NT-proBNP, pg/mL	158.50 (52.00, 615.00)	38.00 (32.00, 94.00)	203.00 (83.00, 854.00)	< 0.001*
RHC data				
sPAP, mmHg	56.00 (38.00, 77.00)	26.00 (23.75, 27.25)	63.00 (44.00, 79.00)	< 0.001*
dPAP, mmHg	18.00 (13.00, 24.50)	9.00 (7.00, 12.00)	20.00 (15.00, 25.00)	< 0.001*
mPAP, mmHg	31.00 (23.00, 41.00)	15.50 (14.00, 17.00)	35.00 (26.00, 43.50)	< 0.001*
PAWP, mmHg	11.00 (9.00, 12.00)	10.00 (7.00, 12.00)	11.00 (9.00, 12.00)	0.286
mRAP, mmHg	4.00 (2.00, 6.75)	3.00 (2.00, 4.00)	5.00 (3.00, 7.00)	0.001*
mRVP, mmHg	19.00 (14.00, 27.00)	11.00 (9.50, 12.00)	21.00 (16.00, 29.00)	< 0.001*
PVR, Wood units	6.25 (2.77, 9.86)	1.19 (0.72, 1.94)	7.29 (4.27, 10.74)	< 0.001*
PVRI, Wood units·m^2^	10.43 (5.01, 17.27)	2.18 (1.31, 3.00)	12.43 (7.79, 18.84)	< 0.001*
CO, L/min	3.65 (2.98, 4.44)	4.94 (4.17, 5.83)	3.47 (2.88, 4.11)	< 0.001*
CI, L/min/m^2^	2.16 (1.77, 2.50)	2.76 (2.29, 3.14)	2.05 (1.71, 2.36)	< 0.001*
SvO_2_, %	70.40 (65.40, 74.80)	77.00 (73.20, 78.20)	69.45 (64.35, 73.50)	< 0.001*

Cardiopulmonary comorbidity consisted of pulmonary comorbidities, including chronic obstructive pulmonary disease, asthma, interstitial lung disease, and cardiac diseases such as coronary heart disease and hypertension

*CTEPD* chronic thromboembolic pulmonary disease, *CTEPH* chronic thromboembolic pulmonary hypertension, *BMI* body mass index, *PTE* pulmonary thromboembolism, *WHO-FC* World Health Organization functional class, *6MWD* 6-min walk distance, *PaO*_*2*_ partial pressure of arterial oxygen, *NT pro-BNP* N-terminal pro-brain natriuretic peptide, *RHC* right heart catheterization, *mPAP* mean pulmonary arterial pressure, *PAWP* pulmonary arterial wedge pressure, *mRAP* mean right atrial pressure, *mRVP* mean right ventricular pressure, *PVR* pulmonary vascular resistance, *PVRI* pulmonary vascular resistance index, *CO* cardiac output, *CI* cardiac index, *SvO*_*2*_ mixed venous oxygen saturation

* Statistically significant

Compared with patients with CTEPD, those with CTEPH were significantly older (60 vs. 48.5 years, *p* = 0.004) and had a longer disease duration (376.00 vs. 216.50 days, *p* = 0.027). No significant differences were observed between groups in terms of sex, BMI, smoking status, VTE or cancer history. However, patients with CTEPH exhibited significantly lower 6MWD, impaired oxygenation index, and elevated levels of D-dimer and NT-proBNP (all *p* < 0.05), as well as increased mPAP and PVR than those with CTEPD. In contrast, CO, CI, and SvO_2_ were significantly lower in the CTEPH group.

### Perfusion parameters between CTEPD and CTEPH

PerAIDE successfully segmented and quantified normal, reduced, and defective perfusion regions in 31 ± 3 s—substantially faster than manual radiologist assessments, which required 15 ± 4 min (*p* < 0.001). The malperfused regions identified by PerAIDE showed excellent agreement with manual PBV scores, with an ICC of 0.778 (95% confidence interval (CI): 0.715–0.828). Patients with CTEPH had significantly higher proportions of perfusion defects than those with CTEPD (Table 2; 0.35 vs. 0.29, *p* < 0.001), while patients with CTEPD showed a greater proportion of reduced perfusion areas (0.36 vs. 0.30, *p* < 0.001). No significant difference was observed in the proportion of normally perfused regions between the two groups (*p* = 0.256). In addition, PBV scores were significantly higher in the CTEPH group (PBV score 1: 0.33 vs. 0.17, *p* < 0.001; PBV score 2: 0.31 vs. 0.14, *p* < 0.001), as shown in Table 2.

**Table 2:** Comparison of perfusion parameters between CTEPD and CTEPH

Perfusion parameters	All patients (*n* = 183)	CTEPD (*n* = 32)	CTEPH (*n* = 151)	*p*-value
PerAIDE				
The percentage of normal perfusion	0.34 (0.32, 0.38)	0.36 (0.31, 0.39)	0.34 (0.32, 0.37)	0.256
The percentage of perfusion defects	0.34 (0.30, 0.39)	0.29 (0.24, 0.32)	0.35 (0.32, 0.40)	< 0.001*
The percentage of reduced perfusion	0.31 (0.26, 0.36)	0.36 (0.29, 0.39)	0.30 (0.24, 0.35)	< 0.001*
Perfusion defects/reduced perfusion	1.07 (0.84, 1.50)	0.79 (0.62, 0.99)	1.14 (0.89, 1.55)	< 0.001*
PBV score 1	0.31 (0.22, 0.43)	0.17 (0.08, 0.25)	0.33 (0.25, 0.44)	< 0.001*
PBV score 2	0.28 (0.19, 0.42)	0.14 (0.10, 0.23)	0.31 (0.22, 0.42)	< 0.001*

*CTEPD* chronic thromboembolic pulmonary disease, *CTEPH* chronic thromboembolic pulmonary hypertension, *AI* artificial intelligence, *PBV* perfusion blood volume

* Statistically significant

### Correlation between PerAIDE-derived perfusion parameters and hemodynamics

**Fig. 4:**
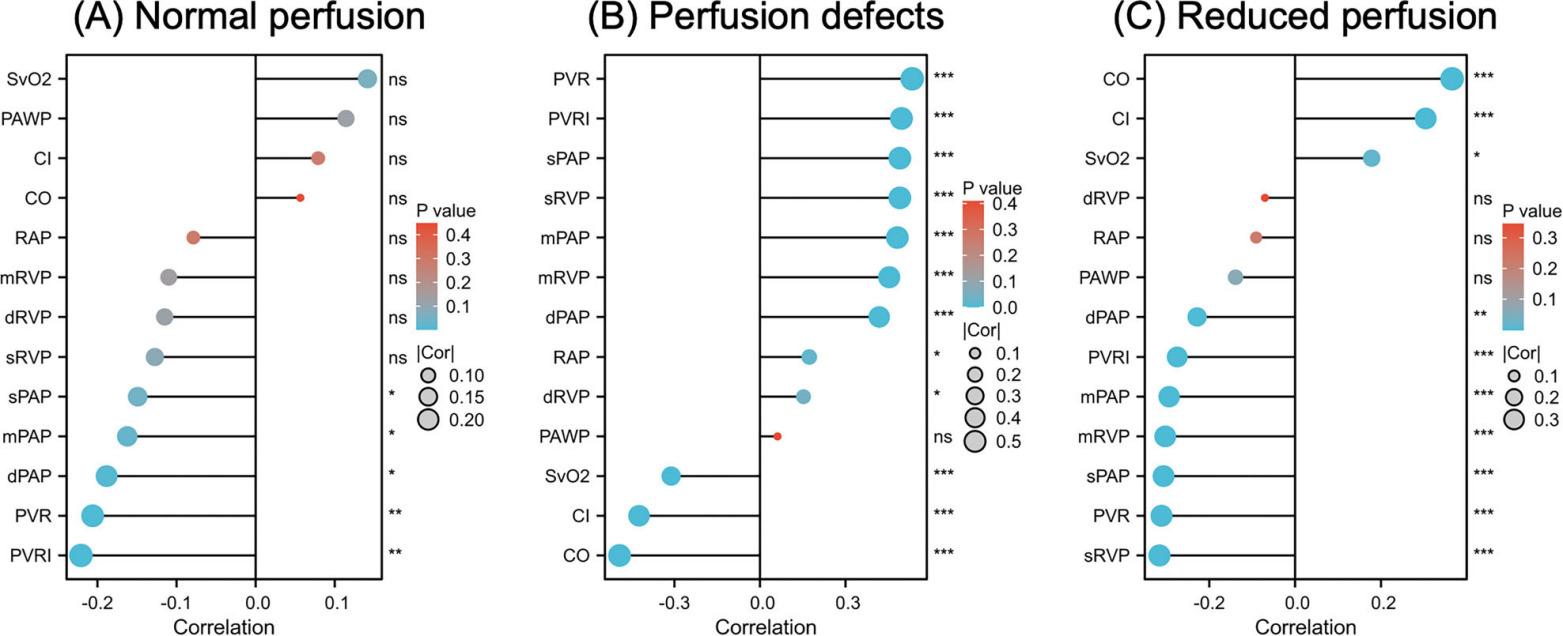
The correlation between PerAIDE-based perfusion parameters and RHC metrics. (**A**) Correlation between normal perfusion parameter and hemodynamics; (**B**) Correlation between perfusion defects parameter and hemodynamics; (**C**) Correlation between reduced perfusion parameter and hemodynamics. ns: no significance; * *p* < 0.05; *** *p* < 0.01. AI, artificial intelligence; RHC, right heart catheterization; mPAP, the mean pulmonary artery pressure; mRVP, mean right ventricular pressure; mRAP, mean right atrial pressure; PAWP, pulmonary arterial wedge pressure; PVR, pulmonary vascular resistance; PVRI, pulmonary vascular resistance index; SvO_2_, mixed venous oxygen saturation; CO, cardiac output; CI, cardiac index; PerAIDE, pulmonary perfusion artificial intelligence automatic quantification analysis system based on DE-CTPA

PerAIDE-derived perfusion parameters demonstrated significant correlations with both hemodynamic and clinical variables (Fig. 4). Perfusion defects showed a moderate positive correlation with PVR (ρ = 0.534) and a weak positive correlation with mPAP (ρ = 0.482) while demonstrating inverse correlations with CO (ρ = −0.492) and SvO_2_ (ρ = −0.311) (all *p* < 0.01). Conversely, reduced perfusion areas displayed weak positive correlations with CO (ρ = 0.366) and SvO_2_ (ρ = 0.178), and weak negative correlations with sRVP (ρ = −0.316), sPAP (ρ = −0.306), and PVR (ρ = −0.311).

Clinically, perfusion defects were inversely associated with functional capacity and gas exchange metrics, including 6MWD (ρ = −0.284) and oxygenation index (ρ = −0.441), and positively correlated with NT-proBNP levels (ρ = 0.428) (Fig. S2). In contrast, reduced perfusion regions demonstrated positive correlations with both 6MWD (ρ = 0.235) and oxygenation index (ρ = 0.315).

### Predictive performance of perfusion parameters for CPE and poor WHO-FC

**Fig. 5:**
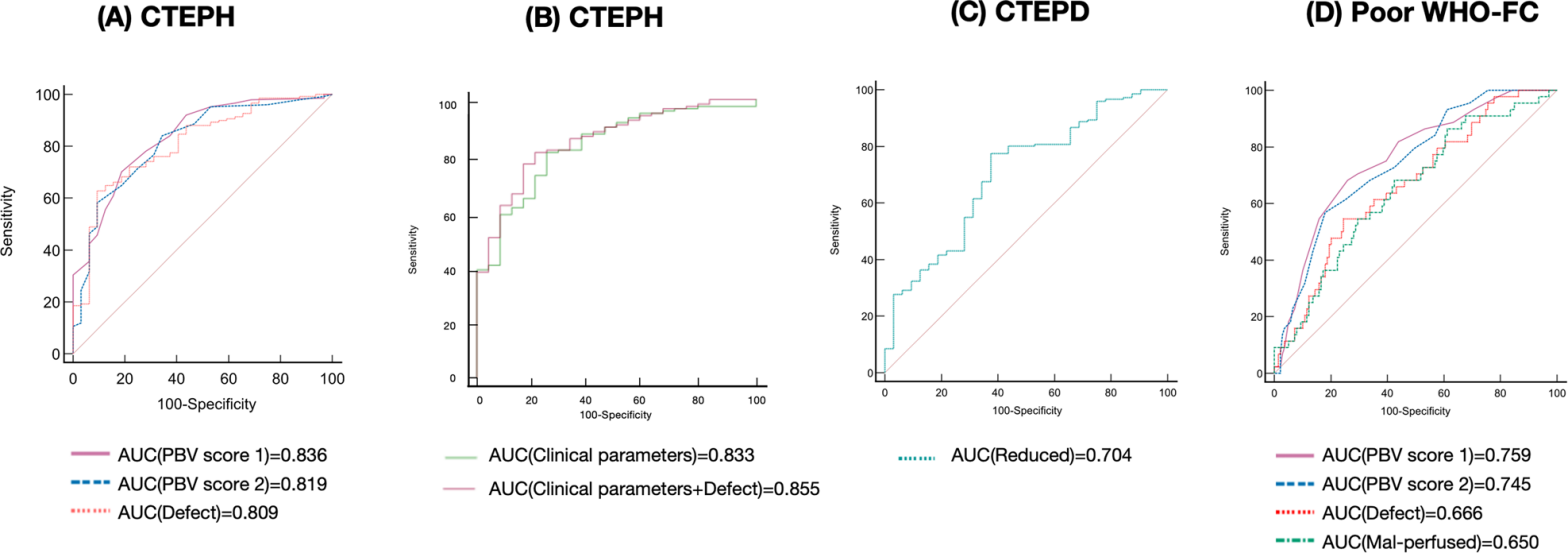
The ROC curves of perfusion and clinical parameters in identifying CTEPH, CTEPD, and poor WHO-FC. (**A**) ROC curves for diagnosing CTEPH using PBV scores and PerAIDE-derived perfusion defects; (**B**) ROC curves for diagnosing CTEPH using clinical parameters with and without PerAIDE-derived perfusion defects; (**C**) The ROC curve for diagnosing CTEPD using PerAIDE-derived reduced perfusion; (**D**) ROC curves for predicting poor WHO-FC using PBV scores and PerAIDE-derived perfusion parameters. CTEPH, chronic thromboembolic pulmonary hypertension; CTEPD, chronic thromboembolic pulmonary disease; WHO-FC, World Health Organization functional class; ROC, receiver operating characteristic; PBV, perfusion blood volume

ROC analysis revealed that PerAIDE-derived perfusion defects demonstrated comparable diagnostic performance to radiologists’ PBV scores in differentiating CTEPH from CTEPD. The AUC for PerAIDE-based perfusion defects was 0.809 (95% CI: 0.745–0.863), which was similar to PBV score 1 (AUC: 0.836; 95% CI: 0.774–0.887) and PBV score 2 (AUC: 0.819; 95% CI: 0.755–0.872), with no statistically significant differences between them (*p* > 0.05; Fig. 5A). The optimal diagnostic threshold for PerAIDE-based perfusion defects was 0.34, yielding a specificity of 90.62% and a sensitivity of 62.91% for diagnosing CTEPH.

To further enhance diagnostic accuracy, clinical parameters including 6MWD and NT-proBNP were incorporated. These clinical parameters alone showed an AUC of 0.833 (95% CI: 0.751–0.914) for identifying CTEPH (Fig. 5B). When PerAIDE-derived perfusion defects were added to this clinical model, the combined AUC improved to 0.855 (95% CI: 0.780–0.929). However, DeLong’s test indicated that this improvement was not statistically significant (*p* = 0.45). In addition, reduced perfusion areas quantified by PerAIDE were able to distinguish CTEPD with an AUC of 0.704 (95% CI: 0.632–0.769; Fig. 5C).

A total of 44 cases (24.04%) were classified as having poor WHO-FC III or IV. ROC analysis showed that PBV score 1 and PBV score 2 could accurately identify patients with poor WHO-FC, with AUCs of 0.759 (95% CI: 0.690–0.819) and 0.745 (95% CI: 0.675–0.806), respectively (Fig. 5D). Similarly, the AUCs for PerAIDE-based perfusion defect and malperfused regions were 0.666 (95% CI: 0.592–0.734) and 0.650 (95% CI: 0.576–0.719), respectively. No significant differences were observed between these methods (*p* = 0.83).

### Lung perfusion parameters before and after balloon pulmonary angioplasty (BPA) treatment

**Fig. 6:**
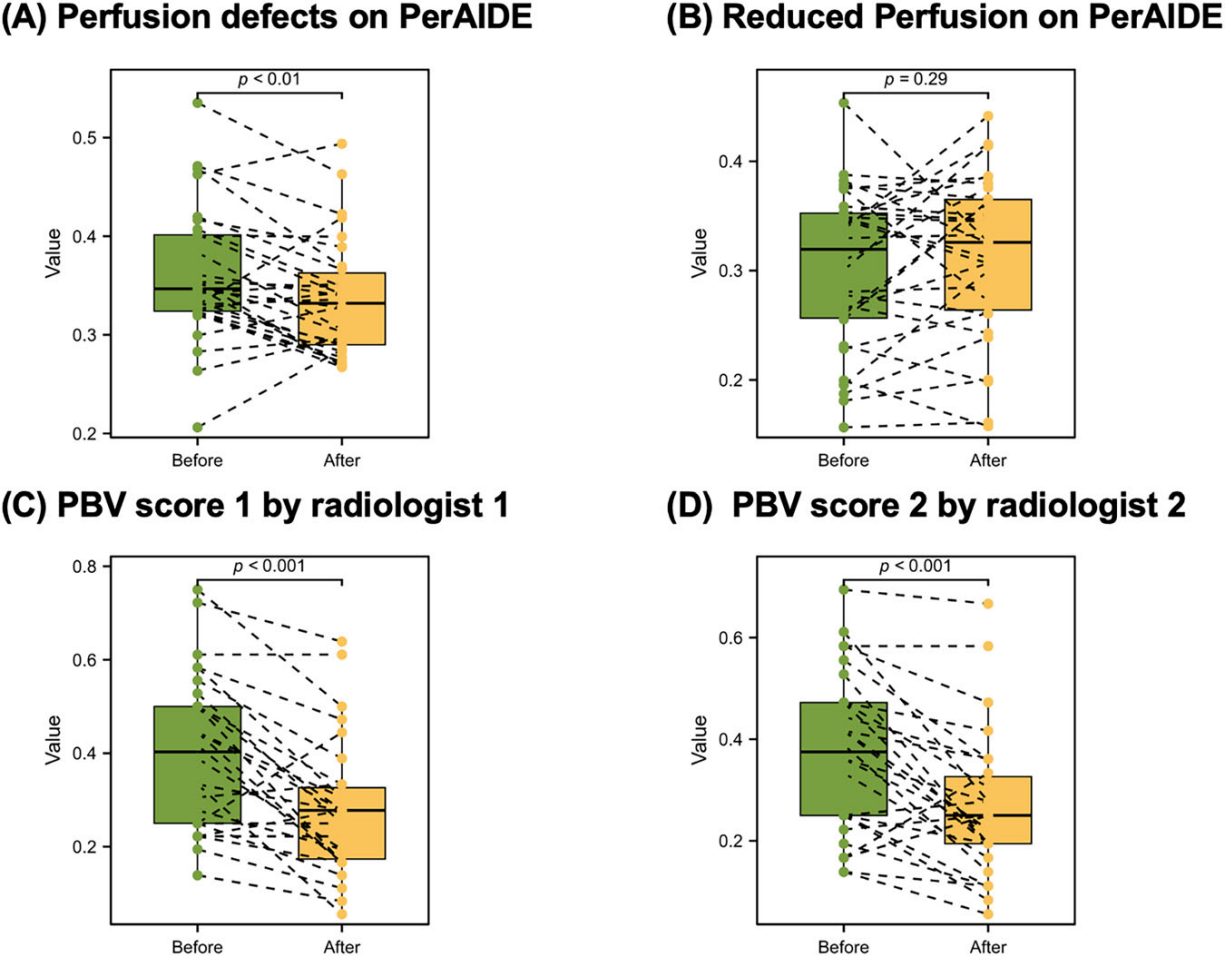
Changes in lung perfusion parameters before and after BPA treatment. (**A**) Changes of PerAIDE-derived perfusion defects; (**B**) Changes of PerAIDE-derived reduced perfusion; (**C**) Changes of PBV score 1; (**D**) Changes of PBV score 2. BPA, balloon pulmonary angioplasty; PBV, perfusion blood volume; PerAIDE, pulmonary perfusion artificial intelligence automatic quantification analysis system based on DE-CTPA

In a subset of 30 patients who underwent follow-up DE-CTPA 3 months after BPA, PerAIDE analysis revealed a significant reduction in perfusion defects (0.36 ± 0.07 vs. 0.33 ± 0.06; *p* < 0.01; Fig. 6), while reduced perfusion areas remained stable (*p* = 0.29). Similarly, radiologist-assessed PBV scores demonstrated significant improvements post-BPA (score 1: 0.40 ± 0.16 to 0.28 ± 0.14; score 2: 0.37 ± 0.16 to 0.26 ± 0.14; both *p* < 0.01), indicating measurable enhancements in lung perfusion following intervention.

## Discussion

In this study, we developed PerAIDE, an automated pulmonary perfusion quantification system utilizing DE-CTPA. PerAIDE integrates a U-Net deep learning architecture with an adaptive thresholding algorithm to facilitate objective and rapid assessment of CPE. The principal findings are summarized in the Graphical Abstract: (1) PerAIDE can more effectively quantify perfusion on DE-CTPA and shows good consistency with manual PBV scoring by radiologists; (2) The perfusion defect percentages positively correlate with PVR and mPAP, whereas reduced perfusion percentages negatively correlate with these hemodynamic parameters; (3) CTEPH is characterized by extensive perfusion defects, while CTEPD is characterized by more reduced perfusion and fewer perfusion defects; (4) Perfusion defects quantified by PerAIDE closely reflect clinical severity and correlate with treatment response in patients with CPE.

PerAIDE’s design leverages the encoder-decoder structure of U-Net with skip connections to achieve accurate lung segmentation. The adaptive thresholding algorithm adjusts segmentation parameters dynamically based on real-time feedback [17–19], optimizing the balance between sensitivity and specificity [15]. This methodological approach aligns with successful applications of similar algorithms in medical imaging, such as quantification of hemorrhagic transformation in acute ischemic stroke CT scans [11] and infarct core segmentation [12]. PerAIDE not only matches radiologists in accuracy for classifying perfusion patterns on DE-CTPA but also reduces processing time dramatically, underscoring its potential as a clinically valuable tool for rapid perfusion evaluation.

PBV measurements derived from DE-CTPA provide direct insight into pulmonary blood flow and are instrumental for assessing the circulatory status [20]. Accumulating evidence supports PBV quantification as a prognostic marker across a spectrum of pulmonary conditions, including APE, chronic obstructive pulmonary disease, and even coronavirus disease 2019 (COVID-19) [21–23]. Previous semi-automated methods segmented lung regions into normal and malperfused areas but lacked differentiation between reduced perfusion and true perfusion defects [24]. To our knowledge, this study is the first to introduce the concept of pulmonary reduced perfusion—akin to the ischemic penumbra concept in cerebral ischemia—and to elucidate its clinical relevance in CTEPD and CTEPH.

Our findings are consistent with earlier studies demonstrating significant correlations between perfusion metrics and invasive hemodynamic parameters in CPE. Notably, when perfusion defects quantified by PerAIDE exceed 34%, the probability of concomitant PH increases substantially. Prior research has similarly reported positive associations between PBV scores and invasive hemodynamics, supporting their role as indicators of CTEPH severity [16, 25–27]. Interestingly, this study demonstrated that the reduced perfusion percentage is inversely associated with hemodynamic parameters in patients with CPE and can effectively predict the presence of CTEPD. In clinical practice, differentiating CTEPH from CTEPD primarily hinges on the detection of resting PH. However, CTEPH often manifests with subtle or nonspecific symptoms in its early stages, making diagnosis challenging. Confirmation typically requires invasive RHC, yet some patients may be reluctant to undergo this procedure early in the disease course. As a result, diagnosis is frequently delayed—by an average of 14.1 months—leading to a postponement of appropriate treatment [28]. Given these challenges, PerAIDE-derived perfusion parameters may serve as a valuable noninvasive tool to support clinicians in identifying patients who warrant further evaluation with RHC.

Using PerAIDE, we identified distinct perfusion phenotypes between CTEPH and CTEPD. Patients with CTEPH showed extensive perfusion defects, while patients with CTEPD primarily displayed reduced perfusion areas with minimal defects. These distinct imaging phenotypes provide valuable insight into the pathophysiological differences underlying these conditions. Evidence suggests that the severity of perfusion defects reflects the extent of microvascular remodeling and damage [29]. For instance, a study of systemic sclerosis patients demonstrated that those with abnormal pulmonary perfusion, despite minimal or no overt lung infiltrates on CT, experienced worse dyspnea and reduced exercise tolerance, indicative of microvascular pathology [30]. Radiological differentiation of CTEPD and CTEPH primarily depends on identifying such microvascular involvement [31, 32]. The perfusion patterns detected by DE-CTPA in this study may therefore represent a radiographic correlation with microvascular disease severity, accounting for the observed differences in clinical and hemodynamic profiles.

This study demonstrates that the PerAIDE-based perfusion defect metric correlates significantly with cardiopulmonary function in patients with CPE and can assist in clinical risk stratification. Moreover, incorporating PerAIDE-derived perfusion defect measurements into existing clinical parameters enhances the predictive accuracy for diagnosing CTEPH to a certain extent. PBV scores obtained from DE-CTPA correlate with right heart strain and disease severity in patients with APE, highlighting their potential as prognostic indicators [21, 29, 33–38]. In cases where CT imaging fails to detect clear thrombi, the extent of pulmonary perfusion defects on DE-CTPA is associated with all-cause mortality risk, underscoring the critical clinical importance of pulmonary perfusion assessment [39]. Additionally, our findings suggest that PerAIDE-derived perfusion defects act as markers of hypoxemia. Similar observations have been reported in patients with other pulmonary conditions such as pulmonary emphysema, lung cancer, and COVID-19 [22, 23, 40].

Furthermore, PerAIDE-based perfusion defects show significant improvement after BPA treatment, correlate strongly with clinical efficacy, and have promising utility as a follow-up tool for assessing treatment response and determining BPA endpoints [41]. Koike et al reported comparable results, demonstrating that improvements in PBV reflect enhanced pulmonary perfusion and correlate positively with mPAP, PVR, and 6MWD [42].

Nevertheless, our study has several limitations. First, although conducted at a single center, it represents the largest CPE cohort to date; however, multicenter validation of PerAIDE’s performance is necessary. Second, the current AI segmentation approach is limited to whole-lung analysis—future developments should incorporate lobar and segmental quantification to better guide BPA planning and evaluate treatment efficacy. Third, the study did not integrate morphological imaging data or long-term clinical outcomes, highlighting the need for future research to combine radiomic features with extended follow-up to improve clinical applicability.

## Conclusion

PerAIDE provides accurate quantification of pulmonary perfusion abnormalities effectively and shows a strong correlation with the clinical severity of CPE, underscoring its potential utility in both initial evaluation and longitudinal follow-up. Furthermore, the distinct perfusion patterns identified by PerAIDE in CTEPD and CTEPH offer novel insights into their underlying pathophysiological differences. These imaging-based phenotypes may help elucidate the mechanisms driving the progression from perfusion abnormalities to overt PH in CTEPH, thereby supporting more targeted diagnostic and therapeutic strategies.

## Supplementary information

ELECTRONIC SUPPLEMENTARY MATERIAL

## Data Availability

The datasets generated during and/or analyzed during the current study are available from the corresponding author upon reasonable request.

## Abbreviations

6MWD: 6-min walk distance
AI: Artificial intelligence
AUC: Area under the curve
BMI: Body mass index
CI: Cardiac index
CO: Cardiac output
COVID-19: Coronavirus 2019
CPE: Chronic pulmonary thromboembolism
CTEPD: Chronic thromboembolic pulmonary disease
CTEPH: Chronic thromboembolic pulmonary hypertension
DE-CTPA: Dual-energy computed tomography pulmonary angiography
ICC: Interclass correlation coefficient
mPAP: Mean pulmonary artery pressure
mRAP: Mean right atrial pressure
PBV: Perfusion blood volume
PerAIDE: Pulmonary perfusion Artificial Intelligence automatic quantification analysis system based on DE-CTPA
PH: Pulmonary hypertension
PVR: Pulmonary vascular resistance
RHC: Right heart catheterization
ROC: Receiver operating characteristic
sPAP: Systolic pulmonary artery pressure
sRVP: Systolic right ventricular pressure
SvO_2_: Mixed venous oxygen saturation
V/Q: Ventilation/perfusion
VTE: Venous thromboembolism
WHO-FC: World Health Organization functional class
