# Supplementary material for: Development of a lung perfusion automated quantitative model based on dual-energy CT pulmonary angiography in patients with chronic pulmonary thromboembolism
Source: Insights Imaging. 2025 Aug 18;16:182. doi: 10.1186/s13244-025-02067-6 (PMC12361028; doi:10.1186/s13244-025-02067-6)
Supplement: Supplementary file 1 — ELECTRONIC SUPPLEMENTARY MATERIAL [file 13244_2025_2067_MOESM1_ESM.pdf]

# Development of a Lung Perfusion Automated Quantitative Model Based on Dual-Energy CT Pulmonary Angiography in Patients with Chronic Pulmonary Thromboembolism

## ELECTRONIC SUPPLEMENTARY MATERIAL


**Part 1 Materials**

| | | |
|---|---|---|
| **Material S1** | The detailed information of the PerAIDE development | Page 2 |

**Part 2 Figures**

| | | |
|---|---|---|
| **Figure S1** | Illustration of PBV Scores in Typical Cases | Page 6 |
| **Figure S2** | The correlation between PerAIDE-based perfusion parameters and clinical characteristics | Page 7 |

## Part 1 The detailed information of the PerAIDE development

### *1.1 Data preprocessing*

DE-CTPA images of all patients were anonymized, extracted as Digital Imaging and Communications in Medicine (DICOM) format. The image preprocessing stage plays a vital role in enhancing the quality and consistency of both CT and PBV images. First, we apply noise reduction techniques to minimize artifacts and improve image clarity. For CT images, we employ adaptive bilateral filtering methods[1], which effectively preserve edge details while reducing noise. A similar approach is applied to PBV images, with additional consideration given to their functional nature. Next, we perform intensity normalization to ensure consistent grayscale ranges across all images, facilitating more reliable subsequent analysis. This step is particularly crucial for PBV images, as it enables more accurate comparisons of perfusion values between different lung regions and across patients.

We also apply geometric corrections to address any distortions in the images, ensuring anatomical accuracy. This includes correcting for patient movement and respiratory artifacts, which are removed through connected component analysis. Components smaller than 5% of the largest connected component are discarded to eliminate non-lung tissues. Finally, histogram equalization is applied separately to each lung to enhance local contrast. Specifically, we use contrast limited adaptive histogram equalization (CLAHE) [2] with a clip limit of 2.0 and a tile grid size of 8 × 8. This step improves the visibility of lung structures in CT images and enhances the differentiation of perfusion levels in PBV images.

### *1.2 Image segmentation*

For the accurate delineation of lung regions from the preprocessed CT images, we utilize a deep learning-based approach, specifically employing a

modified U-Net architecture, optimized for lung segmentation tasks to achieve robust and precise results. Our U-Net model *(Figure 2)* consists of an encoder path for feature extraction and a decoder path for accurate localization, with skip connections to preserve fine-grained details. The encoder path of U-Net includes four consecutive downsampling blocks. Each block starts with two 3 × 3 convolutional layers, followed by batch normalization and ReLU activation. Batch normalization stabilizes the learning process by mitigating internal covariate shift. After the convolutional layers, a 2 × 2 max pooling operation with stride 2 is applied for downsampling, effectively doubling the number of feature channels. The initial block starts with 64 feature channels, which double at each downsampling step, reaching 512 channels at the bottleneck. To prevent overfitting—particularly important due to the limited size of medical imaging datasets—a dropout layer with a rate of 0.5 is implemented at the bottleneck. This is followed by two 3 × 3 convolutional layers with batch normalization and ReLU activation, maintaining 512 feature channels.

The decoder path mirrors the encoder, comprising four upsampling blocks. Each block begins with a 2 × 2 transposed convolution (deconvolution) that reduces the number of feature channels by half and doubles the spatial dimensions. A key feature of U-Net is the use of skip connections, where upsampled features are concatenated with corresponding feature maps from the encoder path. The final layer of the network is a 1 × 1 convolutional layer with a sigmoid activation function, producing a probability map for lung segmentation.

The network was trained on 512×512-pixel CT images with a batch size of 16, balancing memory constraints and effective batch normalization. We used the Adam optimizer with an initial learning rate of $1e^{-4}$. The loss function was a combination of Dice loss and binary cross-entropy, providing a balance

between region-based and pixel-wise accuracy. The segmentation results from our U-Net model serve as a vital input for the subsequent stages of our lung function assessment pipeline. They enable accurate registration with PBV images and facilitate the precise quantification of lung perfusion parameters.

### *1.3 Quantification and visualization*

The quantification and visualization process begins with accurately segmented and registered CT and PBV images *(Figure 2)*. We apply an adaptive thresholding algorithm to the registered PBV image pixels within the segmented lung regions. This approach can dynamically adjust the thresholds based on local intensity distributions, accounting for variations in perfusion levels across different patients and lung areas. The adaptive method ensures robust classification, even in the presence of heterogeneous perfusion patterns. The algorithm classifies the lung regions into: perfusion defects, reduced perfusion, and normal perfusion. Next, we conduct spatial analysis to refine these classifications, considering neighboring pixels and applying a smoothing filter to minimize noise and isolated misclassifications. Rigid registration of the CT and PBV images is then performed to ensure precise alignment between the anatomical and perfusion data. Quantitative metrics are calculated for both the left and right lungs, including the percentage and absolute volume of each perfusion character. We also compute a perfusion score which accounts for both the extent and severity of perfusion abnormalities, providing a concise assessment of lung perfusion. In addition, we generate comprehensive reports that combine the quantitative metrics with key visualizations, facilitating efficient clinical decision-making and improving communication among healthcare professionals. The complete code for constructing PerAIDE can be found on the GitHub website (https://github.com/chengyong/dectlung)

## References


1. Paris S, Kornprobst P, Tumblin J, Durand F: Bilateral Filtering: Theory and Applications. *Foundations and Trends® in Computer Graphics and Vision* 2009, 4(1):1-73.
2. Vidhya GR, Ramesh H: Effectiveness of Contrast Limited Adaptive Histogram Equalization Technique on Multispectral Satellite Imagery. *Proceedings of the International Conference on Video and Image Processing* 2017.

**Part 2 Figures**

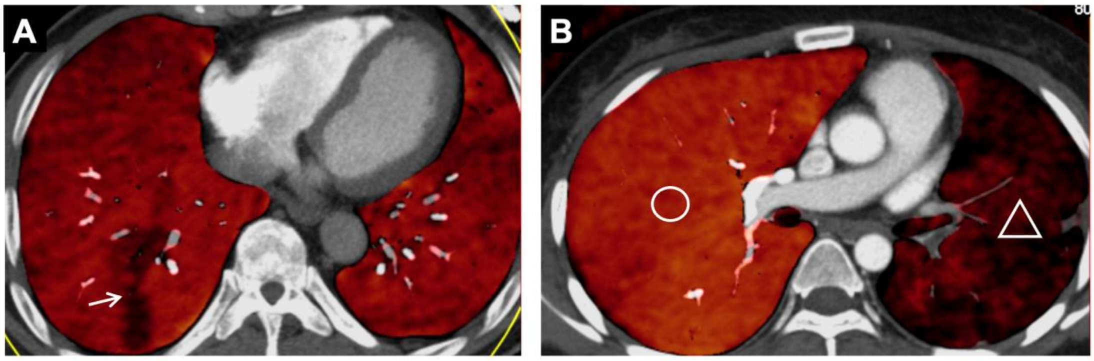


**Figure S1.** Illustration of PBV Scores in Typical Cases. In Figure S1. A, the black area indicated by the white arrow represents the filling defect area in the posterior basal segment of the right lower lobe. As its volume is less than 50% of the segment, the score is 1. In Figure S1. B, the circular red area in the right lung shows good blood perfusion in all segments with no filling defect areas, so the score is 0. However, the dark red and black areas in the left lung (triangle area) indicate that the volume of perfusion defect areas in each segment exceeds 50% of the segment, resulting in a score of 2.

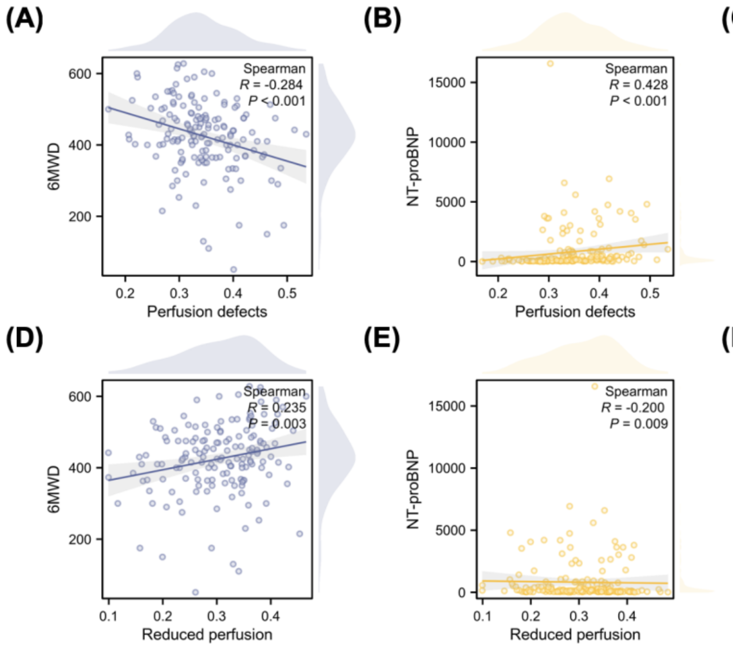


**Figure S2.** The correlation between PerAIDE-based perfusion parameters and clinical characteristics. Abbreviations: 6MWD, six-minute walk distance.
